# Experimental validation that human microbiome phages use alternative genetic coding

**DOI:** 10.1038/s41467-022-32979-6

**Published:** 2022-09-29

**Authors:** Samantha L. Peters, Adair L. Borges, Richard J. Giannone, Michael J. Morowitz, Jillian F. Banfield, Robert L. Hettich

**Affiliations:** 1 Biosciences Division, Oak Ridge National Laboratory, Oak Ridge, TN USA; 2 Graduate School of Genome Science and Technology, The University of Tennessee, Knoxville, Knoxville, TN USA; 3 Innovative Genomics Institute, University of California, Berkeley, CA USA; 4 Environmental Science, Policy and Management, University of California, Berkeley, CA USA; 5 Department of Surgery, University of Pittsburgh School of Medicine, Pittsburgh, PA USA; 6 Earth and Planetary Science, University of California, Berkeley, CA USA; 7 Lawrence Berkeley National Laboratory, Berkeley, CA USA; 8 Chan Zuckerberg Biohub, San Francisco, CA USA

**Keywords:** Phage biology, Viral genetics

## Abstract

Previous bioinformatic analyses of metagenomic data have indicated that bacteriophages can use genetic codes different from those of their host bacteria. In particular, reassignment of stop codon TAG to glutamine (a variation known as ‘genetic code 15’) has been predicted. Here, we use LC-MS/MS-based metaproteomics of human fecal samples to provide experimental evidence of the use of genetic code 15 in two crAss-like phages. Furthermore, the proteomic data from several phage structural proteins supports the reassignment of the TAG stop codon to glutamine late in the phage infection cycle. Thus, our work experimentally validates the expression of genetic code 15 in human microbiome phages.

## Abstract (web summary)

Previous bioinformatic analyses have indicated that bacteriophages can use genetic codes different from those of their host bacteria. Here, Peters et al. use metaproteomics to provide experimental evidence of reassignment of stop codon TAG to glutamine in phages found in the human gut microbiome.

## Introduction

Bacteriophages modulate the composition of microbial communities through the selective predation of bacteria, alteration of host metabolism, and redistribution of cellular lysis products in the environment during the infection process^1^. Despite their recognized importance as components of ecosystem dynamics, phages remain one of the most poorly understood members of microbiomes^2,3^ due to limitations of the methodologies used to study them. Fundamental questions remain regarding how phages interact with and redirect, the translation systems of their host bacteria throughout their infection process.

There is evidence from metagenomic studies that some phages appear to use the bacterial ribosome to translate their proteins using both the standard and an alternative code^1,4–8^. In these phages, proteins that require a stop codon to be read as an amino acid are thought to be only translated late in the infection cycle after code switch machinery has been deployed^5^. Some phages are predicted to reassign the normal stop codon TAG to be translated as glutamine (Q), and others reassign the TGA stop codon to tryptophan (W). This phenomenon appears to be common in human and animal microbiomes^1,4–8^ and is particularly prevalent in phages that infect Firmicutes and Bacteroidetes^3^.

If not recognized, stop codon reassignment can limit our ability to identify phages in metagenome sequences and restrict our understanding of phage gene inventories. Specifically, incorrect code usage leads to low predicted coding densities, truncated gene products, and genes predicted in incorrect reading frames. Alternative code choices can be tested to determine if they restore full-length open reading frames, and the amino acid to which a stop codon is reassigned can be predicted based on amino acid alignments with homologous proteins from related phages. Direct proteomic confirmation of gene predictions of stop codon reassignment has been primarily restricted to bacteria^9,10^, but predictions for this type of alternative coding event in phages have never been experimentally validated. Nonetheless, validation of alternative genetic coding is crucial for the accurate understanding of the translation of gene products, and the utilization of experimental evidence provided by proteomics for genome reannotation based on atypical codon usage has been reported in several studies^11–13^. For example, proteomics-based systematic characterization of the N-termini of proteins validated more than 60 non-canonical translation initiation codons in the *Deinococcus deserti*^14^. Bioinformatic studies have inferred the use of genetic code 15 in some bacteriophages^4,5,8^ and the protist *Iotanema spirale*^15^. However, there are no reports validating the expression of this alternate code, and it has been actively rejected in the summary of genetic codes recognized by NCBI (https://www.ncbi.nlm.nih.gov/Taxonomy/Utils/wprintgc.cgi). In this work, we use LC-MS/MS-based metaproteomics to demonstrate expressional evidence for stop codon reassignment in two crAss-like phages found in the human gut and experimentally validate the expression of genetic code 15.

## Results

Our previous metagenomic study^16^ identified two unrelated human microbiome samples that each contained an abundant crAss-like phage. The adult sample contained a 191 kilobase crAss-like phage genome with the potential to circularize, and the infant sample had a 94 kilobase crAss-like phage genome, which was curated to completion (Supplementary Fig. 1). These samples were prioritized for metaproteomic measurements to address two key questions: (1) can proteins of phages be detected in the presence of highly abundant bacterial, human, and dietary proteins, and (2) can phage proteins be detected that confirm the expression of alternative genetic code 15?

To answer these questions, paired metagenomic and metaproteomic measurements were conducted on fecal samples containing abundant crAss-like phages from one infant and one adult. Metagenomic data indicated these phages are predicted to use genetic code 15, based on the increased coding density observed with translations using genetic code 15 relative to genetic code 11. To ensure accurate peptide identifications from the metaproteomes, assembled metagenomic data from the same samples were used to generate databases that included phage proteins that were predicted using either the standard genetic code 11 (TAG→stop) or alternative genetic code 15 (TAG→Q), as well as all other bacterial proteins in the sample, the human reference proteome, and proteins commonly found as contaminants.

Phages contribute a relatively small proportion of proteinaceous biomass in fecal samples, making detecting their proteins by shotgun proteomics particularly challenging. In fact, initial measurements of the fecal samples detected no phage proteins. Thus, a combination of centrifugation and filtration-based enrichment techniques was employed to enrich phage particles and their proteins irrespective of the phage’s physical size. Fecal phage enrichment strategies for proteomics typically separate phage particles from other microbial biomass in the sample with a 0.2 μm filter, under the assumption that phage particles will be smaller in size than bacterial cells^17^. Previous work has shown alternatively coding phages have genome sizes, and presumably corresponding physical sizes, that range from very small to very large^5^. Thus, we developed a workflow to first separate phage particles, regardless of size, from bacterial cells in the sample using a low-speed centrifugation step (Supplementary Fig. 2). The resulting supernatant is then passed through a 0.8 μm filter to further remove non-proteinaceous debris. The eluted material is finally passed over a 300 kDa MWCO filter to capture intact phage particles on top of the filter while passing through highly abundant human proteins from epithelial cells that were lysed during the initial thawing and homogenization steps. In addition, the pellet from the low-speed centrifugation step can be further processed to examine phage proteins that are present inside the host bacterial cells. Overall, this enrichment strategy enables the successful detection of low abundance phage proteins in the presence of highly abundant proteins from the human host and bacteria. The LC-MS/MS data was searched against the comprehensive sample-matched databases that included phage proteins predicted using either code 11 or code 15. Identified peptides were evaluated codon by codon to determine whether translation using standard or alternative genetic code was appropriate. To complement the database search strategy, de novo peptide sequencing, which derives peptide sequence information directly from the MS/MS spectra, was incorporated into the traditional database search workflow to provide a database-independent confirmation of phage translation that is agnostic to the translation code used for gene predictions.

**Fig. 1:**
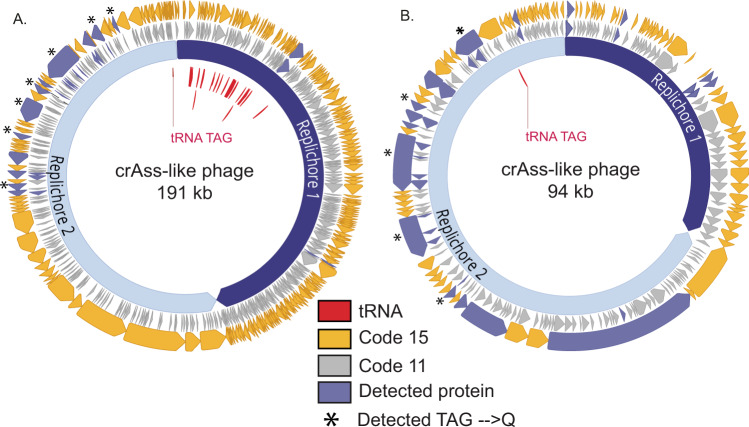
Proteomic detection of alternatively coded proteins from two phage genomes. L2_026_000M1_scaffold_35 (**A**) and L3_063_250G2_scaffold_974_curated (**B**) are alternatively coded crAss-like phages. Prediction of genes in code 11 (middle gray ring) leads to gene fragmentation and low coding density, while gene prediction in code 15 (outer yellow ring) restores open reading frames. Genes with detected peptide evidence are colored purple. Some detected peptides contain glutamines encoded by reassigned TAG codons, and these genes with these validated recoding events are marked with stars. Suppressor tRNAs (red labels) are predicted to suppress translation termination at recoded TAG stop codons. Individual replichores were identified based on GC skew patterns indicative of bidirectional replication.

Database searching of the phage-enriched fraction of the samples yielded 173 phage-specific peptides in total, with peptide-level false discovery rates at <1%. These peptides mapped to 16 and 14 phage proteins in the infant and adult samples, respectively. In addition, numerous peptides and proteins from bacteria and humans were identified (Supplementary Data 1, 2, 4, 5). Many of the phage peptides identified by database searching were further supported by de novo sequencing tags. Roughly half of the identified phage peptides in each sample mapped only to proteins predicted using genetic code 15. Figure 1 shows the genome maps of the target phages in each sample, with the locations of predicted and detected proteins using either code 11 or code 15 translation. Some of the proteins identified with code 15 predictions were annotated as structural proteins, including capsid, portal, and tail-associated proteins (Supplementary Data 3 and 6), while the remaining proteins were unannotated. The detection of mostly late infection structural proteins was expected based on the enrichment for viral-like particles employed for sample preparation.

Across all identified phage proteins in these samples, 67% of genetic code 15 proteins could be confidently annotated, while only 34% of standard genetic code 11 proteins could be confidently annotated. As incorrect code prediction leads to genes predicted in incorrect reading frames and truncated gene products, this discrepancy in annotations levels is alarming. It does emphasize the need for correct code usage during gene predictions in order to accurately catalog phage gene inventories, as very few insights on biological function can be elucidated from incorrectly, and poorly, annotated genomes.

**Fig. 2:**
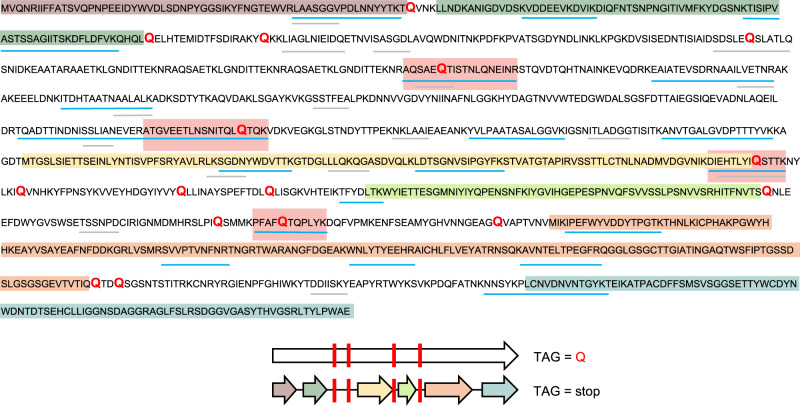
Protein sequence coverage map of alternative code phage tail-related protein. Highlighted sections of the code 15 predicted protein sequence (top) show the corresponding proteins that would have been predicted using standard code 11 (predicted open reading frames), also depicted in the graphical representation (bottom). Blue lines illustrate regions covered by tryptic peptides identified through LC-MS/MS database matching, whereas gray lines represent regions of the predicted protein sequence with matching de novo sequence tag coverage. Red text in the sequence indicates the location of glutamine residues from reassigned stop codons. Red boxes on the sequence coverage map and red bars on graphical representation indicate the recoded glutamine residues with peptides identified through database searching.

Figure 2 shows the protein sequence coverage map from the alternatively coded phage tail fiber protein (L3_063_250G2_scaffold_974_curated_39.code15) identified in the infant fecal sample. The region of the phage genome corresponding to this single protein would have constituted six truncated proteins when predicted using the standard code. However, when using code 15, the full-length alternatively coded protein contained 23 peptides identified through database matching, of which, 11 were exclusively identified using code 15. Four peptides, highlighted in red boxes, directly confirm that the TAG stop codon is reassigned to glutamine. The identification of several de novo sequencing tags provides additional evidence of the existence and expression of recoded stop codons in this alternatively coded protein.

**Fig. 3:**
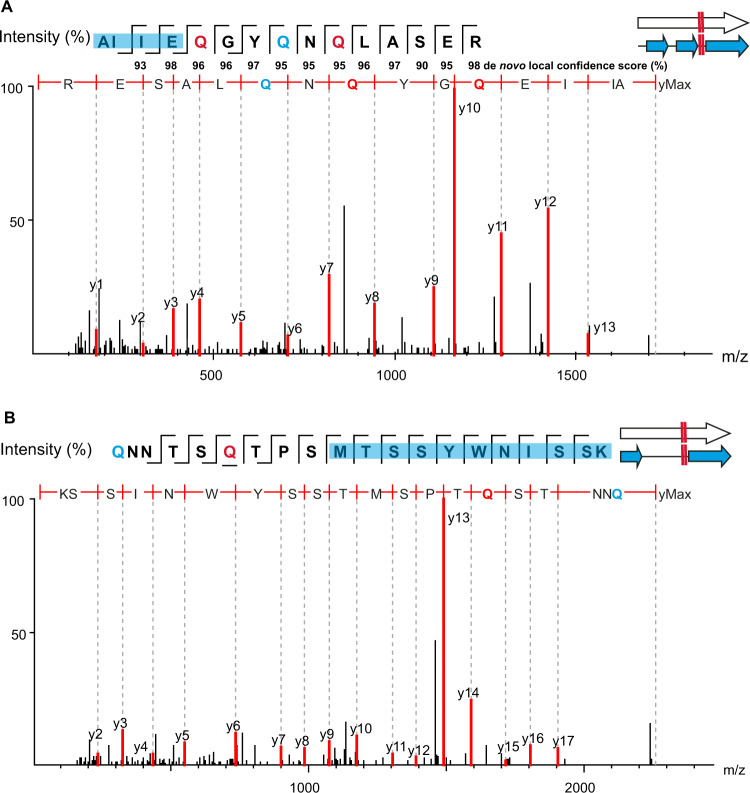
Example MS/MS spectra of alternative coding tryptic peptides. In all panels, red “Q” represents a stop codon that has been reassigned to glutamine using code 15 translation. Blue “Q” represents canonical glutamine residues. Note—the annotated y-ion series are read from the c-terminus to the n-terminus. (top left) Residues highlighted in blue on the amino acid sequence fragmentation ladder depicting b-and y-ion fragmentation series represent portions of the peptide also predicted through code 11 (standard code). **A** Read-through of a standard genetic code 11 stop codon (L2_026_000M1_scaffold_35_232). **B** Read-through of a standard genetic code 11 start codon (L3_063_250G2_scaffold_974_curated_32).

Numerous identified peptides in both the infant and adult fecal samples further substantiate phage reassignment of the TAG stop codon to glutamine. Figure 3 shows two examples of high-quality MS/MS spectra for alternatively coded phage peptides. In both instances, the glutamine residue from the recoded stop codon was positioned in the middle of a tryptic peptide. In the figure, only the direct y-type fragment ion series was chosen for annotation due to their preferential generation in higher-energy C-trap dissociation (HCD) fragmentation during MS/MS measurement^18^.

The peptide in Fig. 3A contains three glutamine residues; one canonical glutamine and two glutamines from recoded stop codons. One of the recoded glutamines was predicted as a stop codon at the end of a protein predicted through standard code translation. With a nearly complete fragmentation ion series, the detected tryptic peptide shows several amino acids flanking this recoded stop codon, covering an amino acid sequence that would not exist in a standard code open reading frame. In addition, a de novo sequencing tag matching nearly the entire length of the database match had high local confidence scores for every amino acid residue, including the recoded glutamines, providing additional support that this peptide, and others like it, do in fact exist (Supplementary Fig. 3). Figure 3B shows a peptide containing a methionine from a predicted start codon using standard code residing in the middle of the peptide sequence in addition to a glutamine from a recoded stop codon. As several amino acids depicted here map to codons upstream of the standard code methionine start codon, this tryptic peptide would not exist if the phage was using standard code translation.

In order to expand the understanding of when these phages might deploy alternate coding, additional LC-MS/MS measurements were conducted on the unenriched fraction of the fecal samples that primarily contained unlysed bacterial cells and host proteins to determine if any additional phage peptides could be detected for early infection proteins that would be present in the host bacterium at the time of sample processing. Measurement yielded the detection of numerous phage peptides in the infant sample, including peptides for three additional proteins that were not identified in the original phage enriched samples. These three proteins included two hypothetical proteins and one ribosomal protein found in a region of the genome predicted to use genetic code 11. We found no evidence of stop codon recoding (code 15) in any of these new peptides in the sample fraction expected to contain intracellular early infection phage proteins. This supports the current hypothesis that alternatively coded phages employ stop codon recoding to prevent premature expression of structural and lytic phage genes at inappropriate times during the phage infection cycle.

Finally, as genetic code 15 only utilizes ATG as a start codon for translation initiation, confirmation of expression of this start codon was necessary to validate this genetic code. Supplementary Fig. 4 shows an example of direct peptide sequencing of a peptide containing a methionine from the ATG start codon for a genetic code 15 predicted protein, confirming translation initiation at this site in the genome. To confirm that alternative start codons were not being utilized, additional databases were generated to determine if translation was being initiated upstream of the predicted ATG start codon. Searches with databases that extended the protein-coding sequences several amino acids upstream of the predicted start codons yielded no peptide evidence that translation was occurring upstream of the predicted code 15 open reading frame. These examples provide experimental validation that standard genetic code 11 is not being utilized by the phage in the translation of this region of the genome, and instead, genetic code 15 is being used. In total, there is copious expressional evidence of genetic code 15, including direct evidence of stop codon readthrough and peptides existing outside of genetic code 11 predicted open reading frames, in regions of the genome with increased coding density using genetic code 15 predictions compared to code 11 predictions. There is no peptide evidence of TAG stop codon recoding in genome regions predicted to use standard genetic code 11 based on a similar coding density for each of the genetic codes. This peptide evidence supports the assignments of genetic codes based on relative coding densities for these regions of the genome.

## Discussion

It has been suggested that alternatively coded structural proteins may be a strategy employed by phages to prevent premature expression of structural and lytic phage genes during the replication process^6^. In this study, the combination of metagenomics and metaproteomics confirmed that when it occurs within genes, the TAG stop codon can be translated as glutamine. This not only confirms the expression of code 15, which had never previously been experimentally validated in any biological system but also should help enhance better genomic assemblies and annotations for bacteriophages, thereby providing an experimental framework for characterizing the temporal deployment of phage alternative genetic coding as a regulatory mechanism for infections during the phage life cycle. Direct metaproteomic confirmation of alternative codes has rarely been performed, but it is easy to imagine extending this approach to validate other types of alternative genetic coding, such as the use of alternative start codons or incorporation of non-standard amino acids such as selenocysteine and pyrrolysine. A more complete understanding of how phages modulate the genetic code, likely to ensure appropriate translation of their proteins, may have applications in synthetic biology (e.g., where non-standard reading of codons can be used to create non-biological polymers^19^ and in phage engineering.

## Methods

### Human subjects information

The human fecal samples used in this study were collected with informed consent under a reviewed and approved IRB at the University of Pittsburgh (Dr. Michael Morowitz, lead). The research design and results of this study complies with all relevant ethical regulations.

#### Sample selection

Human adult and infant stool samples were collected and sequenced with short-read shotgun sequencing (NCBI BioProject: PRJNA698986)^16^. One adult and one infant sample prioritized for proteomics here were chosen because they both had alternatively coded crAss-like phages present at high abundance. Phage L2_026_000M1_scaffold_35 is the most abundant genome in the adult sample at 659x sequencing coverage, and the next highest coverage genome is a *Bacteroides vulgatus* genome at 307x coverage. Phage L3_063_250G2_scaffold_974 is the most abundant genome in the infant sample at 4752x sequencing coverage, and the next highest coverage genome is a *Bacteroides vulgatus* genome at 242x coverage.

#### Genome predictions and phage genome curation

Coding sequences were predicted by Prodigal^20^ using standard genetic code 11 and alternative genetic code 15. Code assignments were determined based on the relative coding density of contigs predicted using genetic code 11 or code 15. Coding density was calculated by summing the length of all genes in each contig and dividing that length by the total contig length. Contigs of total length 5–100 kb were assigned to use alternative genetic code 15 if there was an increase of greater than 10% coding density for genetic code 15 predictions relative to genetic code 11 predictions. Contigs ≥ 100 kb in length were required to have a coding density increase of greater than 5% with genetic code 15 predictions relative to genetic code 11 predictions to be assigned to be using genetic code 15. HMMER^21^ (hmmsearch) was used to annotate protein sequences with the PFAM, pVOG, VOG, and TIGRFAM HMM libraries. In some cases, BLAST searches against the NCBI database, and remote homology searches using the HHPred^22^ and Phyre2^23^ web portals were used to augment initial annotations. tRNAs were predicted using tRNAscan-s.e. V.2.0 in general mode^24^. Replichores were identified by calculating GC skew (G−C/G+C) and cumulative GC skew using the iRep package (gc_skew.py)^25^. Genome curation was performed using an established method^26^. Curation and genome figure generation were completed using Geneious Prime® 2021.0.3 (https://www.geneious.com/). Additional databases were generated to determine if the translation was occurring upstream of the predicted genetic code 15 start codons, with the inclusion of several codons upstream of the ATG start codon. When possible, a maximum of thirty amino acids upstream of the predicted start codon were appended to the protein sequence. In most cases, there were in-frame stop codons (TAA, or TGA) upstream of the start codon, so the translations included any amino acids between the upstream stop codons and the predicted start codon, which in many cases was less than 30 amino acids.

#### Sample preparation for LC-MS/MS

100 mg stool sample was resuspended in 1200 μL 100 mM Tris-HCl, pH 8.0, and homogenized with 0.9–2.0 mm stainless steel beads (NextAdvance, part #SSB14B). Homogenized samples were incubated for 60 min before centrifugation at 3000 × *g* for 30 min. After centrifugation, the pre-cleared supernatant was filtered on a 0.8 μm pore size PES filter and the eluted volume was further filtered on a 300 kDa MWCO PES filter (Pall, Omega Membrane 300 K, part # OD300C34). The filtered eluate and the residual proteinaceous biomass remaining on top of the filter (resuspended in 100 mM Tris-HCl, pH 8.0) were collected for downstream processing. Samples were adjusted to 4% (wt:wt) sodium dodecyl sulfate (SDS)/5 mM dithiothreitol and incubated at 95 °C for 10 min. Samples were alkylated with 15 mM iodoacetamide (IAA) for twenty minutes at room temperature in the dark. The crude protein sample volume was processed by the protein aggregation capture (PAC) method^27^. Briefly, 300 μg of magnetic beads (1 micron, SpeedBead Magnetic Carboxylate; GE Healthcare UK) was added to each sample. Samples were then adjusted to 70% acetonitrile to induce protein aggregation. Aggregated proteins were washed on a magnetic rack with 1 mL of 100% acetonitrile, followed by 1 mL of 70% ethanol. The washed proteins were then resuspended in 4% SDS/100 mM Tris-HCl, pH 8.0, and boiled off the magnetic beads at 95 °C for 10 min. Protein amounts were quantified by corrected absorbance (Scopes) at 205 nm (NanoDrop OneC; Thermo Fisher). The cleaned proteins were re-aggregated back on the magnetic beads and washed with 1 mL of 100% acetonitrile and 1 mL of 70% ethanol to remove detergent from samples. The aggregated protein pellet was resuspended in 100 mM Tris-HCl, pH 8.0, and digested with 1:75 (wt:wt) proteomics-grade trypsin (Pierce) overnight at 37 °C and again for four hours the following day. Tryptic peptides were filtered on a 10 kDa MWCO filter plate (AcroPrep Advance, Omega 10 K MWCO) at 12,000 × *g* and adjusted to 0.5% formic acid before quantification by NanoDrop OneC.

#### LC-MS-MS

Digested peptides were analyzed by automated 1D LC-MS/MS analysis using a Vanquish ultra-HPLC (UHPLC) system plumbed directly in- line with a QExactive- Plus mass spectrometer (Thermo Scientific). A trapping column (100 µm inner diameter; packed with 5 µm Kinetex C18 reverse-phase resin (Phenomenex) to 10 cm) was coupled to an in-house-pulled nanospray emitter (75 µm inner diameter; 1.7 µm Kinetex C18 reverse-phase resin (Phenomenex) packed to 15 cm). For each sample, 10 µL of peptides were loaded, desalted, and separated by uHPLC under the following conditions: sample injection followed by 100% solvent A (95% H_2_O, 5% acetonitrile, 0.1% formic acid) from 0 to 30 min to load and desalt, a linear gradient from 0 to 30% solvent B (70% acetonitrile, 30% water, 0.1% formic acid) from 30 to 220 min for separation, and 100% solvent A from 220 to 240 min for column re-equilibration. Eluting peptides were measured and sequenced with a QExactive- Plus MS under the following settings: data-dependent acquisition; mass range 300–1500 m/z; MS and MS/MS resolution 70 and 15 K, respectively; MS/MS loop count 20; isolation window 1.8 m/z; charge exclusion unassigned, 1, 6–8.

#### Proteomics data analysis

MS/MS spectra were interrogated by de novo–assisted database searching^28^ in PEAKS Studio 10.6 (Bioinformatics Solutions) against a custom-built proteome database derived from the combination of the sequenced metagenome-derived predicted proteomes for all contigs except the target phage contigs, the phage proteome predicted in standard code (code 11), and the phage proteome predicted in the alternative code (code 15), the human reference proteome from UniProt (UP000005640), common LC-MS/MS protein contaminants, and reversed-decoy sequences of all proteins in the database. Secondary databases were searched against that included several amino acids upstream of the genetic code 15 start codon to confirm if translation was occurring upstream of the predicted start. In all database searches, the parent and fragment ion mass error tolerances were set to ± 10 ppm and ±0.02 Da, respectively. Peptide spectrum matches (PSMs) were required to be tryptic with semi-specific digestion needed and a maximum of three missed cleavages. Accepted modifications included a fixed modification of carbamidomethylation (+57.02) of cysteine residues and a variable modification of methionine oxidation (+15.99), with a maximum of three variable modifications. A false discovery rate of 1% was applied to accept the peptide and protein sequences, and a minimum of one unique peptide was required to identify a protein. De novo only parameters were left at default settings with average local confidence (ALC) scores of >50% and de novo sequence tags displayed if at least six amino acids were shared with the database sequence. The resulting database-identified peptides and corresponding de novo sequence tags were manually validated to generate the final list of phage peptide sequences present in the sample. A database hit passing the 1% FDR threshold was required for a peptide sequence to be considered as detected. De novo sequence tags were regarded as complementary evidence for the code 15 database-identified peptides to confirm the expression of this code only if the residue local confidence scores of the amino acids in the de novo sequence tag matching the database sequence were greater than 90% confidence for each residue. In the cases where a TAG stop codon was reassigned to glutamine, the glutamine and several flanking amino acids in the de novo sequence tag had to pass the >90% residue local confidence score threshold.

### Reporting summary

Further information on research design is available in the Nature Research Reporting Summary linked to this article.

## Supplementary information

Supplementary Information

Description of Additional Supplementary Files

Supplementary Data 1

Supplementary Data 2

Supplementary Data 3

Supplementary Data 4

Supplementary Data 5

Supplementary Data 6

Reporting Summary

## Data Availability

All raw mass spectra for the metaproteome measurement from this study have been deposited into the ProteomeXchange repository with accession numbers: ProteomeXchange-PXD030388; MassIVE-MSV000088561. The deposited data also includes the custom-built proteome database that includes sequenced metagenome-derived predicted proteomes for all contigs except the target phage contigs, the phage proteome predicted in standard code (code 11), and the phage proteome predicted in the alternative code (code 15), the human reference proteome from UniProt (UP000005640), common LC-MS/MS protein contaminants, and reversed-decoy sequences of all proteins. Metagenomics sequencing reads reported in this paper are available under NCBI BioProject: PRJNA698986; SRA: SRR13622550–SRR13622957. Phage genomes assembled from the metagenome sequencing reads are publicly available through Zenodo (10.5281/zenodo.7016025).
