# Supplementary material for: Experimental validation that human microbiome phages use alternative genetic coding
Source: Nat Commun. 2022 Sep 29;13:5710. doi: 10.1038/s41467-022-32979-6 (PMC9523058; doi:10.1038/s41467-022-32979-6)
Supplement: Supplementary file 1 — Supplementary Information [file 41467_2022_32979_MOESM1_ESM.pdf]

Experimental validation that human microbiome phages use alternative genetic coding

Peters *et al.*

Supplementary Information

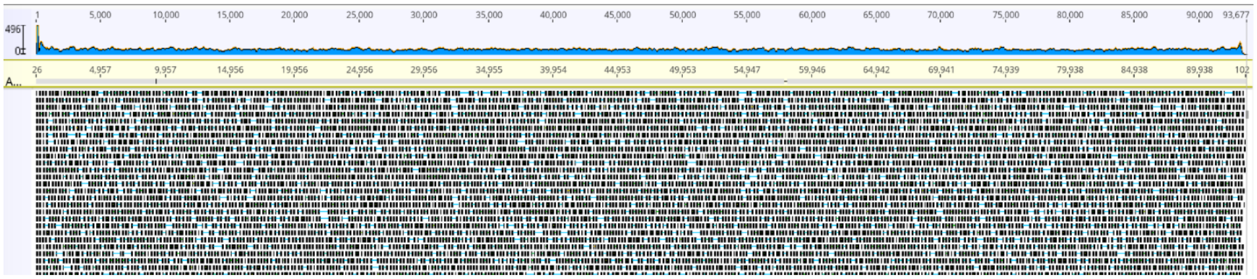

Supplementary Fig. 1. Genome curation and variation. Overview of the final curated genome showing complete and relatively even coverage by paired 150 bp Illumina reads (mean insert size: 391 bp) when reads are mapped, allowing ≤ 2% single nucleotide polymorphisms.

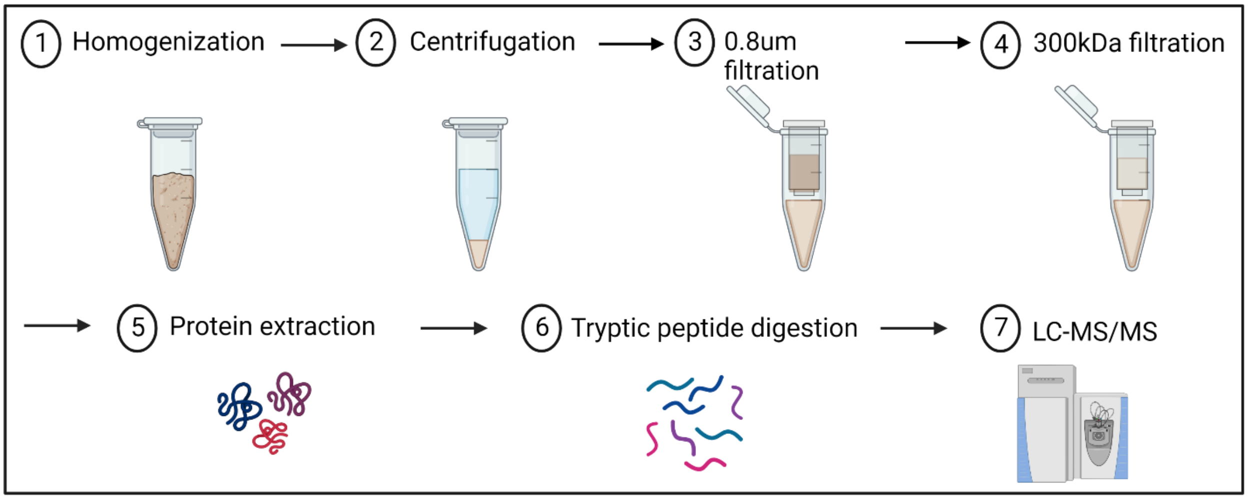

Supplementary Fig. 2. Sample preparation workflow. A combination of low-speed centrifugation and filtration steps were incorporated into the sample preparation workflow for the enrichment of phage particles of various physical sizes from human feces for metaproteomic analysis. Figure generated with Biorender.com.

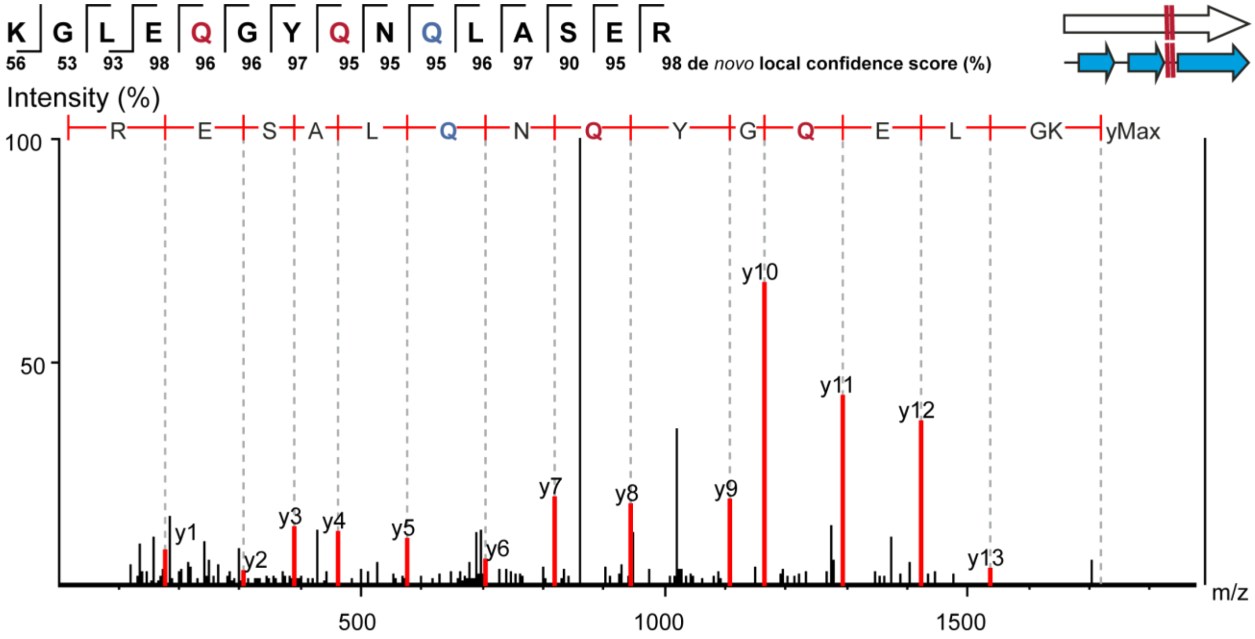

Supplementary Fig. 3. MS/MS spectrum of *de novo* sequence tag of an alternative coded tryptic peptide. The *de novo* sequence tag matches nearly the entire amino acid sequence of the database search identified peptide in Fig. 3A. The canonical glutamine and both glutamine residues from recoded stop codons, as well as flanking residues, have very high *de novo* local confidence scores.

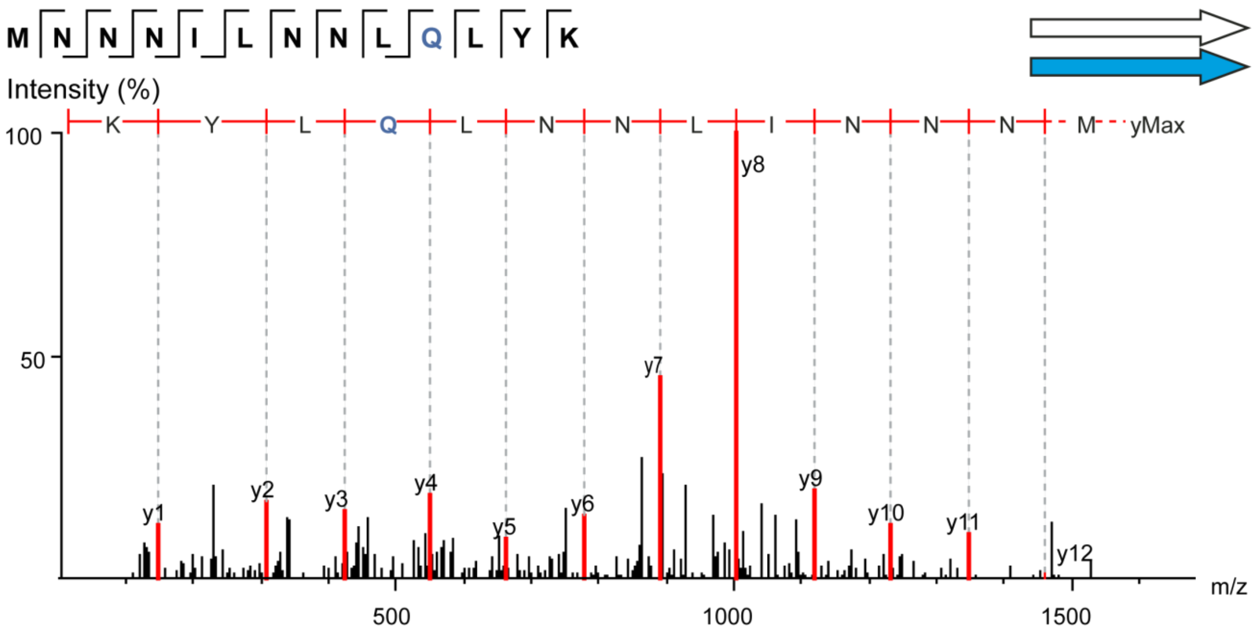

Supplementary Fig. 4. MS/MS spectrum of a tryptic peptide covering a start codon of an alternatively coded protein. The tryptic peptide contains the first thirteen amino acids of protein L3_063_250G2_scaffold_974_curated_25.code15. This demonstrates the methionine from the predicted ATG start codon of genetic code 15 is being translated.
