# Supplementary material for: Experimental validation that human microbiome phages use alternative genetic coding
Source: Nat Commun. 2022 Sep 29;13:5710. doi: 10.1038/s41467-022-32979-6 (PMC9523058; doi:10.1038/s41467-022-32979-6)
Supplement: Supplementary file 2 — Description of Additional Supplementary Files [file 41467_2022_32979_MOESM2_ESM.pdf]

Description of Additional Supplementary Files

File Name: Supplementary Data 1
Description: L2_026_000M1 detected peptides. This table contains all peptides (bacterial, phage, human) from fecal sample L2_026_000M1 identified by LC-MS/MS with a peptide-level false discovery rate (FDR) threshold of 1%.

File Name: Supplementary Data 2
Description: L2_026_000M1 detected proteins. This table contains all proteins (bacterial, phage, human) from fecal sample L2_026_000M1 identified by LC-MS/MS with a protein-level false discovery rate (FDR) threshold of 1% and at least one unique peptide per protein.

File Name: Supplementary Data 3
Description: L2_026_000M1_scaffold_35 detected proteins and peptides. This table contains all detected phage proteins from scaffold L2_026_000M1_scaffold_35. For each protein detected using code 15 predictions (dark green), any corresponding protein predicted using code 11 (light green) with peptide evidence is listed below the code 15 protein. Nested rows below each protein correspond to the peptide analytes detected for the protein. The column "#Peptide analytes" refers to both unmodified and modified versions of the peptide sequence. Peptide rows are coloured to show whether the peptide was found using both code 11 and code 15 predictions (light yellow) or only through code 15 prediction (dark yellow). Red text indicates peptides with at least one sequenced glutamine from a recoded stop codon.

File Name: Supplementary Data 4
Description: L3_063_250G2 detected peptides. This table contains all peptides (bacterial, phage, human) from fecal sample L3_063_250G2 identified by LC-MS/MS with a peptide-level false discovery rate (FDR) threshold of 1%.

File Name: Supplementary Data 5
Description: L3_063_250G2 detected proteins. This table contains all proteins (bacterial, phage, human) from fecal sample L3_063_250G2 identified by LC-MS/MS with a protein-level false discovery rate (FDR) threshold of 1% and at least one unique peptide per protein.

File Name: Supplementary Data 6
Description: L3_063_250G2_scaffold_974 detected proteins and peptides. This table contains all detected phage proteins from scaffold L3_063_250G2_scaffold_974. For each protein detected using code 15 predictions (dark green), any corresponding protein predicted using code 11 (light green) with peptide evidence is listed below the code 15 protein. Nested rows below each protein correspond to the peptide analytes detected for the code 15 protein. The column "#Peptide analytes" refers to both unmodified and modified versions of the peptide sequence. Peptide rows are coloured to show whether the peptide was found using both code 11 and code 15 predictions (light yellow) or only through code 15 prediction (dark yellow). Red text indicates peptides with at least one sequenced glutamine from a recoded stop codon.
