# Supplementary material for: Experimental validation that human microbiome phages use alternative genetic coding
Source: Nat Commun. 2022 Sep 29;13:5710. doi: 10.1038/s41467-022-32979-6 (PMC9523058; doi:10.1038/s41467-022-32979-6)
Supplement: Supplementary file 9 — Reporting Summary [file 41467_2022_32979_MOESM9_ESM.pdf]

nature portfolio

Corresponding author(s): Jillian F. Banfield
Robert L. Hettich

Last updated by author(s): Aug 23, 2022

Reporting Summary

Nature Portfolio wishes to improve the reproducibility of the work that we publish. This form provides structure for consistency and transparency in reporting. For further information on Nature Portfolio policies, see our Editorial Policies and the Editorial Policy Checklist.

Statistics

For all statistical analyses, confirm that the following items are present in the figure legend, table legend, main text, or Methods section.

n/a	Confirmed	
☐	☒	The exact sample size (*n*) for each experimental group/condition, given as a discrete number and unit of measurement
☐	☒	A statement on whether measurements were taken from distinct samples or whether the same sample was measured repeatedly
☒	☐	The statistical test(s) used AND whether they are one- or two-sided<br>*Only common tests should be described solely by name; describe more complex techniques in the Methods section.*
☒	☐	A description of all covariates tested
☒	☐	A description of any assumptions or corrections, such as tests of normality and adjustment for multiple comparisons
☐	☒	A full description of the statistical parameters including central tendency (e.g. means) or other basic estimates (e.g. regression coefficient) AND variation (e.g. standard deviation) or associated estimates of uncertainty (e.g. confidence intervals)
☒	☐	For null hypothesis testing, the test statistic (e.g. *F*, *t*, *r*) with confidence intervals, effect sizes, degrees of freedom and *P* value noted<br>*Give P values as exact values whenever suitable.*
☒	☐	For Bayesian analysis, information on the choice of priors and Markov chain Monte Carlo settings
☒	☐	For hierarchical and complex designs, identification of the appropriate level for tests and full reporting of outcomes
☒	☐	Estimates of effect sizes (e.g. Cohen's *d*, Pearson's *r*), indicating how they were calculated

Our web collection on statistics for biologists contains articles on many of the points above.

Software and code

Policy information about availability of computer code

Data collection	No software was used to collect data in our study.
Data analysis	Prodigal V2.6.3<br>HMMER 3.3 (hmmsearch)<br>HHsuite v3.0.3<br>Phyre2<br>tRNAscan-s.e. V.2.0<br>iRep package V1.10 (gc_skew.py)<br>Geneious Prime® 2021.0.3 (https://www.geneious.com/)<br>PEAKS Studio 10.6 (Bioinformatics Solutions)<br>Protein-Protein BLAST 2.12.0

For manuscripts utilizing custom algorithms or software that are central to the research but not yet described in published literature, software must be made available to editors and reviewers. We strongly encourage code deposition in a community repository (e.g. GitHub). See the Nature Portfolio guidelines for submitting code & software for further information.

Data

Policy information about availability of data

All manuscripts must include a data availability statement. This statement should provide the following information, where applicable:

- Accession codes, unique identifiers, or web links for publicly available datasets
- A description of any restrictions on data availability
- For clinical datasets or third party data, please ensure that the statement adheres to our policy

All raw mass spectra for the metaproteome measurement from this study have been deposited into the ProteomeXchange repository with accession numbers: ProteomeXchange-PXD030388; MassIVE-MSV000088561. The deposited data also includes the custom-built proteome database that includes sequenced metagenome-derived predicted proteomes for all contigs except the target phage contigs, the phage proteome predicted in standard code (code 11), and the phage proteome predicted in the alternative code (code 15), the human reference proteome from UniProt (UP000005640), common LC-MS/MS protein contaminants, and reversed-decoy sequences of all proteins.
Genomes are publicly available as an analysis project via ggKbase (https://ggkbase.berkeley.edu/Alternatively_coded_phage_proteomics/organisms).

Human research participants

Policy information about studies involving human research participants and Sex and Gender in Research.

Reporting on sex and gender	Information related to the sex and gender of the subjects was not collected. Fecal samples used in the present study were selected from knowledge of the presence of near-complete phage genomes of interest from a larger study by co-author Michael Morowitz. Therefore, sex and gender-based analysis was not relevant for the current study.
Population characteristics	N.A. - samples were selected from adult and infant stool samples already collected for a related study. Specific patient ages were not collected for the present study.
Recruitment	N.A. - the human fecal samples used in the present study were selected from samples already collected for a related study.
Ethics oversight	The human fecal samples used were collected with informed consent under a reviewed and approved IRB at the University of Pittsburgh.

Note that full information on the approval of the study protocol must also be provided in the manuscript.

Field-specific reporting

Please select the one below that is the best fit for your research. If you are not sure, read the appropriate sections before making your selection.

- [x] Life sciences
- [ ] Behavioural & social sciences
- [ ] Ecological, evolutionary & environmental sciences

For a reference copy of the document with all sections, see nature.com/documents/nr-reporting-summary-flat.pdf

Life sciences study design

All studies must disclose on these points even when the disclosure is negative.

Sample size	No sample size calculation was performed to determine sample size. This study was designed to capture protein expression of uncultivated gut bacteriophages which use genetic codes that differ from their bacterial hosts. Samples were selected based on two criteria: (1) the presence of target phage genomes and (2) if at least 100mg of stool was available for processing.
Data exclusions	No data was excluded.
Replication	Each fecal sample contained limited biomass and allowed for only one extraction and enrichment, so sample reproducibility cannot be confirmed. However, both fractions (filter residual and flow-through) of the phage enrichment were collected and measured.
Randomization	No randomization was performed due to the limited number of samples.
Blinding	The investigators were not blinded during experiments and outcome assessment. Blinding was not relevant to the study due to the nature of the study since samples were selected for LC-MS/MS measurements based on metagenomic evidence of phage presence.

Reporting for specific materials, systems and methods

We require information from authors about some types of materials, experimental systems and methods used in many studies. Here, indicate whether each material, system or method listed is relevant to your study. If you are not sure if a list item applies to your research, read the appropriate section before selecting a response.

Materials & experimental systems

n/a	Involved in the study
☒	☐ Antibodies
☒	☐ Eukaryotic cell lines
☒	☐ Palaeontology and archaeology
☒	☐ Animals and other organisms
☒	☐ Clinical data
☒	☐ Dual use research of concern

Methods

n/a	Involved in the study
☒	☐ ChIP-seq
☒	☐ Flow cytometry
☒	☐ MRI-based neuroimaging
